# Effectiveness of Carboplatin and Paclitaxel as First- and Second-Line Treatment in 61 Patients with Metastatic Melanoma

**DOI:** 10.1371/journal.pone.0016882

**Published:** 2011-02-16

**Authors:** Annette Pflugfelder, Thomas K. Eigentler, Ulrike Keim, Benjamin Weide, Ulrike Leiter, Kristian Ikenberg, Mark Berneburg, Claus Garbe

**Affiliations:** Department of Dermatology, Center for Dermatooncology, University Hospital Tuebingen, Tuebingen, Germany; Children's Hospital & Harvard Medical School, United States of America

## Abstract

**Background:**

Patients with metastatic melanoma have a very unfavorable prognosis with few therapeutic options. Based on previous promising experiences within a clinical trial involving carboplatin and paclitaxel a series of advanced metastatic melanoma patients were treated with this combination.

**Methods:**

Data of all patients with cutaneous metastatic melanoma treated with carboplatin and paclitaxel (CP) at our institution between October 2005 and December 2007 were retrospectively evaluated. For all patients a once-every-3-weeks dose-intensified regimen was used. Overall and progression free survival were calculated using the method of Kaplan and Meier. Tumour response was evaluated according to RECIST criteria.

**Results:**

61 patients with cutaneous metastatic melanoma were treated with CP. 20 patients (85% M1c) received CP as first-line treatment, 41 patients (90.2% M1c) had received at least one prior systemic therapy for metastatic disease. Main toxicities were myelosuppression, fatigue and peripheral neuropathy. Partial responses were noted in 4.9% of patients, stable disease in 23% of patients. No complete response was observed. Median progression free survival was 10 weeks. Median overall survival was 31 weeks. Response, progression-free and overall survival were equivalent in first- and second-line patients. 60 patients of 61 died after a median follow up of 7 months. Median overall survival differed for patients with controlled disease (PR+SD) (49 weeks) compared to patients with progressive disease (18 weeks).

**Conclusions:**

Among patients with metastatic melanoma a subgroup achieved disease control under CP therapy which may be associated with a survival benefit. This potential advantage has to be weighed against considerable toxicity. Since response rates and survival were not improved in previously untreated patients compared to pretreated patients, CP should thus not be applied as first-line treatment.

## Introduction

Melanoma is an increasingly common disease, and its incidence still rises in the industrialized countries with white populations. Although primary cutaneous melanomas are frequently curable by surgical excision, metastatic melanoma carries a poor prognosis with a median survival ranging from 6 to 12 months, and has not improved during the last three decades. In the US 8700 patients are expected to die of metastatic melanoma in the year 2010 [1].

Metastatic melanoma is a solid tumour that is relatively resistant to systemic treatment [2]. However, chemotherapy with one or more drugs can produce palliative clinical responses in some patients [3]. Currently only dacarbazine and interleukin-2 have been approved for routine therapy of metastatic melanoma. As the majority of patients progress under this treatment or have only short time responses, there is a strong need for second-line treatment options.

Combined chemotherapy with carboplatin and paclitaxel (CP) is a well established treatment regimen in advanced non-small-cell lung cancer and in advanced ovarian cancer [4]–[8]. Carboplatin replaced cisplatin from previous combined regimens demonstrating equal efficacy and less toxicity [9]. This regimen has been used in order to examine combined effects with sorafenib in solid tumours, and, interestingly, melanoma showed promising responses [10]. Therefore, the combination with sorafenib was studied in comparison to CP alone in metastatic melanoma as second-line treatment. Surprisingly, CP treatment results in a long median progression-free survival of four months but sorafenib did not add additional efficacy. Based on the promising results with CP treatment of metastatic melanoma in this phase III trial, many centers introduced this chemotherapy regimen in the treatment of disseminated melanoma, particularly in the second line treatment situation [11]. We started to treat patients with advanced metastatic melanoma with CP in October 2005.

Our patients received CP in case of tumour progression after one or more prior systemic treatments or in case of primarily rapidly progressive disease. The aim of this retrospective analysis was to investigate the effectiveness of CP in advanced melanoma patients in terms of overall survival and response and to compare the results between first and second line treatment.

## Methods

All patients with advanced metastastic melanoma of cutaneous origin receiving CP at our institution between October 2005 and December 2007 were included. Patients with melanoma of ocular origin were excluded. Approval for this retrospective analysis was obtained by the Ethics commitee Tuebingen, German (approval number 384/2010A). Patient data of our own institution were analyzed anonymously, therefore we did not obtain informed consent. This approach was in accordance with the advice of our ethics committee. Approval for this study was gained retrospectively.

Based on the treatment schedule of the second-line CP plus sorafenib trial all patients received intravenous paclitaxel 225 mg/m^2^ plus intravenous carboplatin at area under curve 6 (AUC 6) on day 1 of a 21-day cycle, with a dose reduction after the fourth cycle to carboplatin AUC 5 and paclitaxel 175/mg/m^2^. Some patients in poor general condition or insufficient myelofunction received a reduced dose from the start of treatment. All patients who received at least one cycle were included in the analysis.

Tumour evaluation was based on CT or PET-CT scans, which were obtained after every 3^rd^ cycle (every 9 weeks). Tumour response was evaluated using Response Evaluation Criteria in Solid Tumours (RECIST) criteria [12]. Best response to treatment was classified as complete response (CR) (no clinical or radiologic evidence of disease), partial response (PR) (30% decrease in the sum of the longest diameter), stable disease, and progression of disease (20% increase in the sum of the longest diameter or new lesion). All response evaluations were independently evaluated by a second radiologist and demonstrated to the interdisciplinary skin tumour board at the University Hospital of Tuebingen, Germany.

Statistical analyses were performed with the statistical software SPSS 15.0 (SPSS Inc., Chicago, Illinois, USA). In order to check comparability, first-line and second-line group of patients were compared for the characteristics gender, age, disease classification, brain metastases, liver metastases, number of organs involved, ECOG and LDH level prior to therapy. Bivariate statistical testing was performed using two-sided Chi-square tests. P-values of less than 0.05 were considered statistically significant. Follow up was measured from start of treatment until death or last date of observation. Progression-free survival (PFS) was defined from start of treatment to first documented disease progression. Overall survival (OS) was defined from the start of treatment to the date of death. Non melanoma related deaths were included as censored events. Survival probabilities were calculated according to Kaplan-Meier and compared with log-rank test statistics [13].

## Results

### Patient characteristics

A total of 61 patients were identified for evaluation. Patient characteristics are shown in Table 1. 20 patients (32.8%) received CP as first-line therapy, 33 patients (54.1%) as second-line therapy, eight patients (13.1%) had more than one previous therapy. The median age at start of treatment was 53 years (range 20–79). Concerning the M-classification 54 patients had M1c disease (88.5%), six patients had M1b disease (9.8%), one patient had unresectable stage IIIC disease (1.6%). 22 patients (36.1%) had brain metastases or a history of brain metastases. Six of these 22 patients were treated by surgery, three by stereotactic radiation, nine patients by whole brain radiotherapy and 4 patients had no additional treatment for their brain metastases. 32 patients (52.5%) had liver metastases. The median number of organ sites involved was four (range 1–7). Only 21 patients (34.4%) had normal Eastern Cooperative Oncology Group (ECOG) performance status (PS) prior therapy.

**Table 1:** Patient characteristics (N = 61).

	All patientsN = 61, n (%)	FirstlinepatientsN = 20, n (%)	Second-linePatientsN = 41, n (%)	p-value
**Sex**				
male	34 (56%)	16 (80%)	18 (44%)	0.008
female	27 (44%)	4 (20%)	23 (56%)	
**Median age (years)**	53(range 20–79)	48.5(range 20–69)	59(range 25–79)	0.011
**Disease classification**				
IIIC	1 (2%)	-	1 (2%)	n.s.
M1b	6 (10%)	3 (15%)	3 (7%)	
M1c	54 (89%)	17 (85%)	37 (90%)	
**Brain metastasis prior to therapy with CP**	22 (36%)	10 (50%)	12 (29%)	n.s.
**LDH**				
upper normal limit	42 (69%)	15 (75%)	27 (66%)	n.s.
normal limit	18 (30%)	5 (15%)	13 (32%)	
**No. of organs involved**				
1 organ	7 (12%)	1 (5%)	6 (15%)	n.s.
2 organs	8 (13%)	1 (5%)	7 (17%)	
3 organs	13 (21%)	4 (20%)	9 (22%)	
4 or more organs	33 (54%)	14 (70%)	19 (46%)	
**ECOG prior therapy**				
0	21 (34%)	7 (35%)	14 (34%)	n.s.
1	21 (34%)	5 (25%)	16 (39%)	
2	15 (25%)	5 (25%)	10 (24%)	
3	3 (5%)	3 (15%)	-	
4	1 (2%)	-	1 (2%)	

### Treatment and toxicity

The majority of patients (82%) received the full dosage at start of CP treatment, 18% received the already reduced level (carboplatin AUC 5 and paclitaxel 175/mg/m^2^). The median number of cycles of therapy delivered was four (range 1 to 20). 13 patients (21.3%) received only one cycle of therapy due to clinical disease progression, intolerability or death.

Dose limiting toxicities (grade III and IV) were myelosuppression and peripheral neuropathy. Other frequent toxicities included alopecia and fatigue. In four patients (6.6%) hypersensitivity reactions to paclitaxel occurred. In all of this four patients a rapid desensitization protocol according to a scheme proposed by Lee et al was used to continue therapy [14].

### Response to treatment

**Figure 1:**
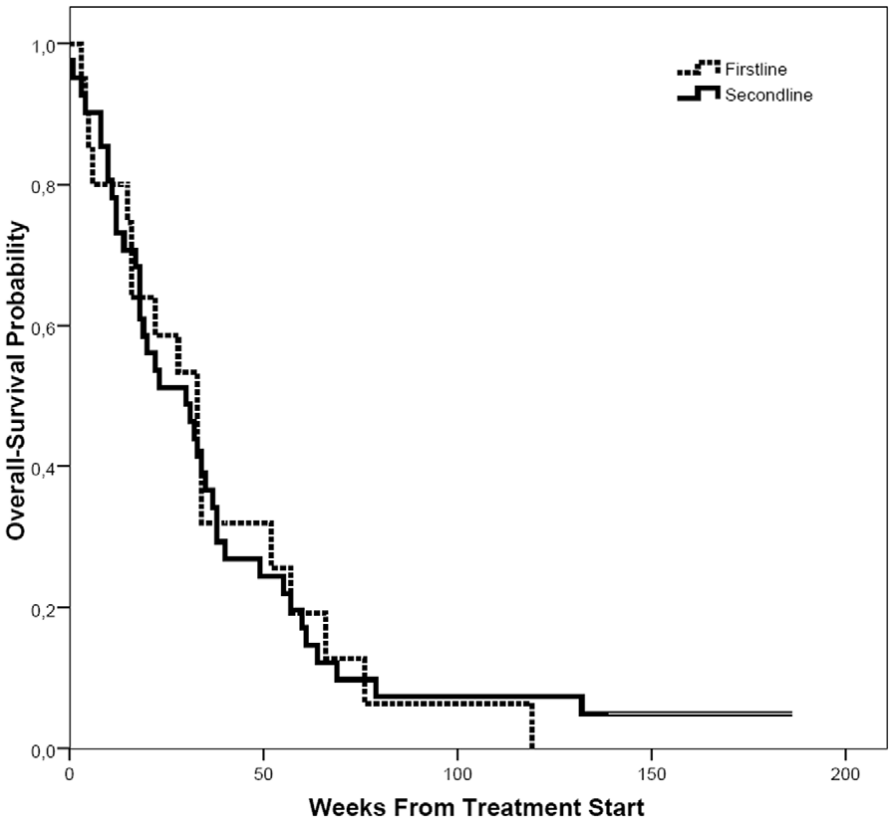
Overall survival. Probability of overall survival after start of treatment in first-line and second-line patients. First-line patients: dotted line, second-line patients: bold line. (p = 0.961).

**Figure 2:**
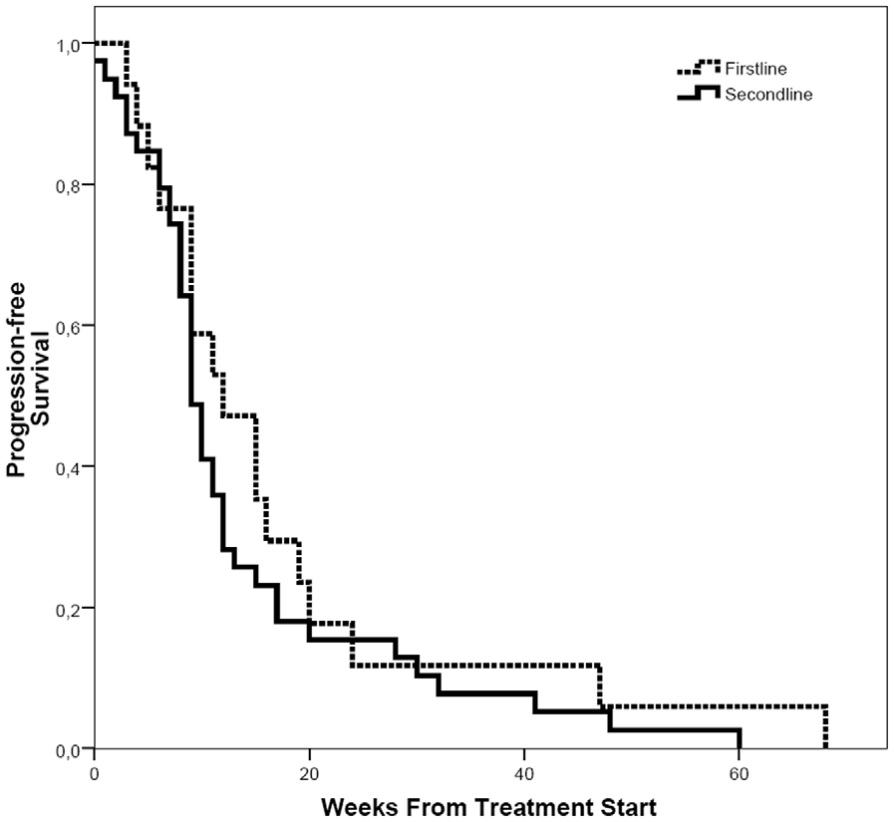
Progression free survival. Probability of progression free survival after start of treatment in first-line and second-line patients. First-line patients: dotted line, second-line patients: bold line. (p = 0.322).

All 61 patients had measurable disease by RECIST criteria and were assessable for response. Response rates are shown in Table 2. Partial response was noted in 4.9% of patients, stable disease in 23% of patients. No complete response was observed. Median progression free survival was 10 weeks (IQR  =  [7, 16]). Median overall survival was 31 weeks (IQR  =  [14, 52]). Response rates as well as progression and disease free survival were equivalent in first- and second-line patients (Figure 1 and 2). Median duration of stability was 22 weeks among the patients with stable disease (n = 14).

**Table 2:** Response rates.

Response	All patientsN = 61n (%)	FirstlinePatientsN = 20n (%)	SecondlinePatientsN = 41n (%)	p-value
**CR**	-	-	-	n.s.
**PR**	3 (4.9%)	1 (5%)	2 (4.9%)	
**SD**	14 (23%)	6 (30%)	8 (20%)	

**Figure 3:**
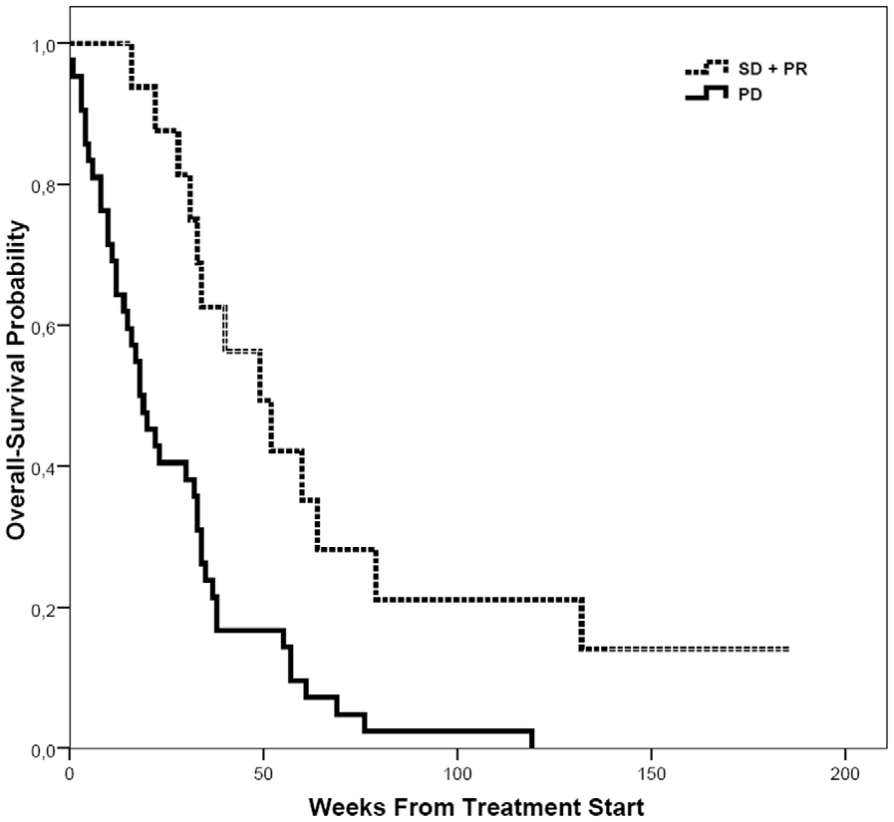
Overall survival in patients with disease control and progressive disease. Probability of overall survival after start of treatment in patients with disease control (SD+PR) and in patients with progressive disease (PD). Patients with disease control: dotted line, patients with progressive disease: bold line. (p = 0.001).

There were no significant differences between patients receiving CP as first-line therapy and second-line therapy regarding S100 levels, LDH levels, ECOG performance status, number of organs involved and presence of brain metastases. But the chemo-naive group of patients included significantly more male (p = 0.008) and young patients (p = 0.011). Among the 22 patients with brain metastases none showed an objective response upon treatment with CP, however stabilisation of disease was observed in 5 patients. After a median follow up of 7 months 60 of 61 patients had died. Median overall survival was 49 weeks (IQR  =  [31, 79]) for patients with controlled disease (partial response and stable disease) compared to 18 weeks (IQR  =  [10. 35]) for patients with progressive disease (p = 0.001) (Figure 3).

There were no significant differences between patients with controlled and progressive disease regarding number of organs involved, presence of brain metastases, presence of liver metastases, age, gender and S100 levels. In contrast, a LDH value over two-fold-upper normal limit at start of CP treatment was significantly associated with progressive disease during therapy (p = 0.009). Decreasing or constant LDH levels under therapy were associated with a prolonged overall survival (p = 0.002).

## Discussion

The present patient collective consisted of patients with clearly progressive metastatic melanoma presenting with widespread metastatic disease. Two third of patients had already received a first-line chemotherapy mainly consisting in dacarbazine-based treatments. First-line patients with extensive metastatic disease were primarily treated with CP because the caring physicians felt that they will be unlikely to respond to dacarbazine. Response to CP treatment was low with five percent of partial responses as well in first-line as in second-line treatment situations. However, 23% of patients achieved stable disease which contributed obviously similarly to prolongation of survival. Thus, temporary disease control was attained in 28% of patients and seemed to be associated with prolongation of survival to 49 weeks as compared to 18 weeks in patients with progressive disease. The CP regimen showed transient disease stabilisations but neither complete responses nor long-term durable responses were accomplished. In one patient the treatment with CP enabled a complete resection of remaining metastases but the patient recurred afterwards. The only patient alive achieved stable disease under CP treatment and was included in a clinical trial with an anti-CTLA 4 antibody, then achieved a CR and has no evidence of disease to date.

Toxicities were manageable in all cases but dose limiting toxicities like myelosuppression and peripheral neuropathy occurred as already described in other tumour entities [15]–[17].

Several studies investigated the combination of CP in metastatic melanoma with different treatment schedules, results and conclusions [11], [18]–[21]. (Table 3) Only 19 patients of our study comply with three main inclusion criteria (ECOG performance status 0 or 1, no cerebral metastases, not more than one prior therapy) of the largest randomized trial by Hauschild et al. It is therefore not possible to compare the two cohorts. The varying results may thus be influenced by different patient selection criteria. The results of our study regarding PFS and OS are similar to a retrospective study published 2005 by Rao et al. [21] Likewise patient characteristics are similar. Both studies reflect the real composition of advanced metastatic melanoma patient cohort in clinical routine.

**Table 3:** Results of published studies on the combination of carboplatin and paclitaxel in patients with metastatic melanoma.

	Current analysis	Hausschild et al.	Perez et al.	Rao et al.	Zimpfer-Rechner et al.	Hodi et al.
**Type of study**	Retro-spective	ProspectivePhase III	ProspectivePhase II	Retro-spective	ProspectivePhase II	ProspectivePhase II
**CP schedule**	every 3 wk	every 3 wk	weekly plus bevacizumab	71% weekly 29% every 3 wk	weekly	every 3 wk
**No of patients**	61	135	53	31	19	15
**Median age y**	53	56	55	59.6	57.6	54
**Prior chemotherapy (% of pat.)**	67.2	100	24.5	100	100	no data
**M1c (% of pat.)**	88.5	69	79	84	no data	39
**CR (% of pat.)**	0	0	0	0	0	0
**PR (% of pat.)**	4.9	11	17	26	0	26
**SD (% of pat.)**	23	51	57	19	16	47
**Median PFS (wk)**	10	17.9	26	12	8	no data
**Median OS (wk)**	31	42	52	31	30	36

In the current study additionally first-line patients were included. Survival curves for OS and PFS were remarkably identical for the cohorts of patients receiving CP as first and second-line treatment. It is noteworthy to mention that only patients with rapidly progressive disease in the first-line situation have been included into this protocol.

The only observed significant difference between patients with controlled and progressive disease was the level of LDH at treatment start. LDH may therefore be considered as a predictive factor for response. It seems to be more likely that factors like tumour load (associated with LDH-level) and number of organs involved but not the aggressiveness and sequence of the applied chemotherapeutic schedules predict treatment responses.

CP therapy in metastatic melanoma has cytostatic effects with achievement of disease control for limited time periods in about one third of patients treated. Complete remissions or durable responses have not been accomplished. It seems not to be a better alternative to dacarbazine treatment in the first-line therapy, and should preferentially be applied as second-line treatment. A response to therapy may be associated with a prolonged overall survival. The indication for CP therapy has to be considered on an individual basis and has to be weighed against considerable toxicity.
